# Reduced Postcapping Period in Honey Bees Surviving *Varroa destructor* by Means of Natural Selection

**DOI:** 10.3390/insects9040149

**Published:** 2018-10-24

**Authors:** Melissa A. Y. Oddie, Bjørn Dahle, Peter Neumann

**Affiliations:** 1 Department of Ecology, Swedish University of Agricultural Sciences, 756 51 Uppsala, Sweden; 2 Department of Animal and Aquacultural Sciences, Norwegian University of Life Sciences, 1430 Ås, Norway; Bjorn.dahle@norbi.no; 3 Norwegian Beekeepers Association, Dyrskuev, 2040 Kløfta, Norway; 4 Institute of Bee Health, Vetsuisse Faculty, University of Bern, 3003 Bern, Switzerland; Peter.neumann@vetsuisse.unibe.ch; 5 Agroscope, Swiss Bee Research Center, 3003 Bern, Switzerland

**Keywords:** *Apis mellifera*, honey bee, mite, natural selection, postcapping period, survivability, *Varroa destructor*

## Abstract

The ectoparasitic mite *Varroa destructor* is a key factor for colony losses in European honey bee subspecies (*Apis mellifera*), but it is also known that some host populations have adapted to the mite by means of natural selection. The role of a shorter host brood postcapping period in reducing mite reproductive success has been investigated in other surviving subspecies, however its role in the adaptation of European honey bee populations has not been addressed. Here, we use a common garden approach to compare the length of the worker brood postcapping period in a Norwegian surviving honey bee population with the postcapping period of a local susceptible population. The data show a significantly shorter postcapping period in the surviving population for ~10% of the brood. Since even small differences in postcapping period can significantly reduce mite reproductive success, this mechanism may well contribute to natural colony survival. It appears most likely that several mechanisms acting together produce the full mite-surviving colony phenotype.

## 1. Introduction

Over the past decade, elevated losses of managed honey bee (*Apis mellifera*) colonies have been reported globally [1,2,3]. There is consensus that the ectoparasitic mite *Varroa destructor* and associated viruses are key factors underlying such losses [4,5,6,7,8,9]. This mite reproduces exclusively in the capped brood cells of the host, and a number of mechanisms have been shown to interfere with mite reproductive success [4].

One of these mechanisms is a shortened postcapping period [4,10]. This trait is of particular interest because of its longstanding association with low mite infestations, e.g., in African honey bee subspecies [11,12]. The postcapping period can vary cell-by-cell within a colony due to factors such as brood genotype as well as the source of the nurse bees both pre and postcapping [13]. This trait has been widely spoken of in the context of breeding programs because of its apparent effectiveness and its heritability [10,14,15]. However, no attempts have yet been made to examine this trait in populations of European honey bees surviving *V. destructor* infestations, populations known to survive without mite treatments by means of natural selection for more than 10 years [16,17,18,19,20]. Since a reduced mite reproductive success seems to be linked to honey bee colony survival in those populations [19,20], the underlying mechanisms are essential for our understanding of the honey bee—*V. destructor* system.

In this study, we measured the impact of genetic lineage on the postcapping period of worker brood in a naturally *V. destructor-*surviving honey bee population with comparison to a local susceptible population. Since a reduced postcapping period has been shown to negatively affect *V. destructor* reproductive success [4], we expect the surviving population to display a shorter postcapping period if this trait impacts survival.

## 2. Materials and Methods

The study was conducted in the Øestlandet region of Norway in July 2017 (local summer), in the range of a local honey bee population naturally surviving *V. destructor* without treatments for >17 years [20]. Mite infestation levels in this population were significantly lower compared to local susceptible colonies and mite reproductive success was reduced by ~30% when compared to the controls [20]. This population, a local Buckfast stock, will from now on be called ‘surviving’. The control colonies chosen were of *A. m. carnica* (Carniolan honey bee) stock obtained from a geographically separate, local conservation area. *A. m. carnica* is a honey bee subspecies known from past studies to be unable to survive *V. destructor* infestations without regular mite treatments [12,13]. This population will from now on be called ‘susceptible’. Five queenright colonies of similar strength (~11 frames of bees) were selected from each of one surviving apiary and one susceptible apiary ~40 km apart. Mite levels at this time of the year were known to be low in all colonies based on bottom-board counts [4] immediately before the start of the experiment (<2 mites per day). From each colony, worker brood frames were chosen with young brood of a similar age (~1–3 days post-hatching) and the frames were labelled individually. The brood on each frame was then carefully mapped using transparent sheets so as to create a brood subset that would be monitored. The 10 test frames were inserted into the same surviving colony in an apiary separate from both donor apiaries for the duration of the uncapped period (~7 days [21]). Surviving and susceptible frames were alternated evenly throughout the box to homogenize humidity and temperature as much as possible across the two groups. The surrogate colony was chosen for its strength and likely ability to rear the added brood. Transference times for both surviving and susceptible frames to the surrogate colony were comparable. The brood was observed daily at 8-h intervals (at 6 a.m., 2 p.m., and 10 p.m.) and each individual cell capped between observation periods was recorded on the transparent sheets with the specific date and time interval. Frames were removed and work was done in a heated room to minimize stress to the surrogate colony. Once ~100 capped cells had been recorded on each frame (after ~48 h of first capped cells and based on brood availability) the frames were moved to a standard, queen-rearing incubator (34.4 °C) and kept there until adult emergence [22]. All frames were moved to the incubator within the span of three days. Emerging workers were checked every 8 h for ~3 days and emergence time interval was recorded for each marked brood cell (N = 1235 total, 530 surviving, 705 susceptible). A χ^2^ two-sample test was used to compare the distributions of emergence times of the two populations so differences across the time bins could be assessed as a whole in a single test.

## 3. Results

**Figure 1:**
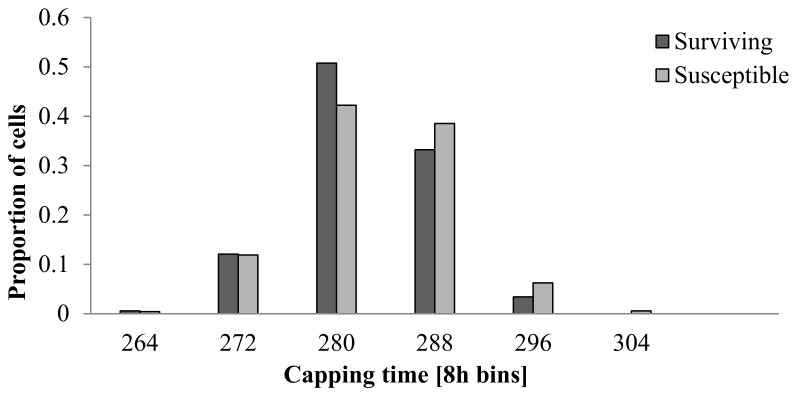
The frequency distribution of postcapping periods in honey bee workers (*Apis mellifera*) from two Norwegian populations, one naturally surviving *Varroa destructor* (dark grey) and one susceptible to *V. destructor* (light grey). Time is accurate within a maximum of 16-h intervals. A significant proportion of surviving bees emerged earlier (χ^2^ = 14.369, df = 5, *p* = 0.013).

There was a significant difference in the duration of the postcapping period between worker bees from surviving and susceptible colonies (Figure 1 and Table 1, χ^2^ = 14.369, df = 5, *p* = 0.013). A higher proportion of *V. destructor*-surviving workers emerged sooner than their susceptible counterparts; approximately 10% more of the surviving brood had emerged after 280 h.

## 4. Discussion

Our data show a significantly reduced postcapping period in honey bee workers from naturally *V. destructor*-surviving colonies of European subspecies. Approximately 10% of the bees from the surviving population emerged in an earlier time window when compared to local, susceptible controls. Since a shorter postcapping period can reduce mite reproductive success [10], this seems to be a mechanism contributing to natural colony survival.

Brood from surviving and susceptible colonies were reared to the capping stage using a common garden approach (same surviving colony and same incubator); therefore, environmental factors that may affect worker postcapping time [21] were similar for both groups. Though genetic variation of nurse bees may also influence the differences between postcapping time, it is the genetic background of the brood [14] that likely explains the observed differences.

While the local surviving population was predominantly “Norwegian Buckfast”, the susceptible colonies were from an *A. m. carnica* conservation area. African subspecies were included in the creation of the Buckfast bees [23], which are known to possess a reduced postcapping period [12]. Therefore, the observed differences in worker postcapping period in this experiment could well reflect a priori genetic differences between the surviving and susceptible bees not resulting from adaptation of the survivors to the selection pressure imposed by *V. destructor*. However, it is well established that the postcapping period can reduce *V. destructor* reproductive success [4]. Therefore, based on our data we cannot confirm whether the observed reduced postcapping period in the surviving population actually constituted a preadaptation to survive mite infestations without treatments or if it evolved within 17 years as an adaptive response.

The observed difference in postcapping period is small. However, a reduction of the postcapping period by a single hour has the potential to reduce *V. destructor* reproductive success by 8.7% [10]. In light of our sample sizes for each population (530 surviving bees and 705 susceptible bees each from five colonies in geographically separate populations), there is likely a significant reduction (between one and 16 h) in the postcapping period within at least ~10% of the surviving brood in this Norwegian population. Shortening the observation interval would likely increase the resolution of the findings. However, since the reproductive success of *V. destructor* in such surviving populations is known to be reduced by at least ~30% in total (populations in France and Sweden [19] and in the population of study [20]), the reduced postcapping period alone is unlikely to explain the mite-surviving colony phenotype in these populations. Instead, it appears as if a range of mechanisms, possibly including brood removal (Varroa Sensitive Hygiene) [24], grooming [25], increased swarming, small colony size [4,26], and other traits may act together to reduce mite reproductive success under the damage threshold in the surviving honey bee populations. Investigations of African honey bee subspecies have demonstrated that postcapping period may not always be linked to reduced reproductive success [27], however in light of the differences in the distribution of postcapping period observed in this study, possible effects cannot be ignored.

## 5. Conclusions

Clearly, the phenotype of naturally *V. destructor*-surviving honey bee colonies is determined by local genotype–environment interactions and the level of mite control that is sufficient in each geographical region [28]. The phenotype likely involves the traits of the honey bee hosts (see above), *V. destructor* mites [16], other interacting pathogens (i.e., viruses [29]), bee forage, climate [28], and beekeeping management [30]. An example: significant differences between local susceptible and surviving Norwegian colonies were found in deformed wing virus titers, which is most likely linked to the striking differences in colony rates of *V. destructor* infestation [31]. It is therefore most likely that adaptations enabling colony survival can differ considerably between populations and that the requirements for survivability in one population may not be the same as the requirements for another. With this in mind, the required level of reduction in postcapping period should not be assumed to be identical for every surviving population and a successful reduction time in a northern environment such as Norway may not be sufficient for populations in more southern climates. In conclusion, we recommend a holistic testing of more populations to finally pinpoint and quantify the contribution of the mechanisms across a wide environmental range that enable honey bee, *A. mellifera*, colonies to survive *V. destructor* infestations without mite treatments.

## Figures and Tables

**Table 1:** Number of worker bees emerging within the designated 8-h time bins. A higher proportion of surviving worker bees emerged at intervals earlier than their susceptible counterparts.

Postcapping Time Interval (h)	Surviving (N = 530)	% of Total Surviving Sample	Susceptible (N = 705)	% of Total Susceptible Sample
264	3	0.6	3	0.4
272	64	12.1	84	11.9
280	269	50.7	298	42.3
288	176	33.2	272	38.6
296	18	3.4	44	6.2
304	0	0.0	4	0.6
